# Formulation and Characterization of Metformin-Loaded Ethosomes for Topical Application to Experimentally Induced Skin Cancer in Mice

**DOI:** 10.3390/ph15060657

**Published:** 2022-05-25

**Authors:** Ibrahim A. Mousa, Taha M. Hammady, Shadeed Gad, Sawsan A. Zaitone, Mohamed El-Sherbiny, Ossama M. Sayed

**Affiliations:** 1 General Authority of Health Care, Ismailia Governorate, Ismailia 11517, Egypt; ibrahim.201@pharm.suez.edu.eg; 2 Department of Pharmaceutics and Industrial Pharmacy, Faculty of Pharmacy, Suez Canal University, Ismailia 41522, Egypt; taha_hamadi@pharm.suez.edu.eg; 3 Department of Pharmacology & Toxicology, Faculty of Pharmacy, Suez Canal University, Ismailia 41522, Egypt; 4 Department of Pharmacology & Toxicology, Faculty of Pharmacy, University of Tabuk, Tabuk 71491, Saudi Arabia; 5 Department of Basic Medical Sciences, College of Medicine, AlMaarefa University, Riyadh P.O. Box 71666, Saudi Arabia; msharbini@mcst.edu.sa; 6 Department of Anatomy, Faculty of Medicine, Mansoura University, Mansoura 3155, Egypt; 7 Department of Pharmaceutics and Industrial Pharmacy, Faculty of Pharmacy, Sinai University, Kantra 41636, Egypt; osama.sayed@su.edu.eg

**Keywords:** experimental skin cancer, entrapment efficacy, ethosomes, metformin, in vitro permeation, zeta potential

## Abstract

To achieve the best treatment of skin cancer, drug penetration inside the deepest layers of the skin is an important scientific interest. We designed an ethosome formulation that serves as a carrier for metformin and measured the in vitro skin permeation. We also aimed to measure the antitumor activity of the optimal ethosomal preparation when applied topically to chemically induced skin cancer in mice. We utilized a statistical Box–Behnken experimental design and applied three variables at three levels: lecithin concentration, cholesterol concentration and a mixture of ethanol and isopropyl alcohol concentrations. All formulations were prepared to calculate the entrapment efficiency %, zeta potential, size of the vesicles and drug release % after 1, 2, 4, 8 and 24 h. The size of the vesicles for the formulations was between 124 ± 14.2 nm and 560 ± 127 nm, while the entrapment efficiency was between 97.8 ± 0.23% and 99.4 ± 0.24%, and the drug release % after 8 h was between 38 ± 0.82% and 66 ± 0.52%. All formulations were introduced into the Box–Behnken software, which selected three formulations; then, one was assigned as an optimal formula. The in vivo antitumor activity of metformin-loaded ethosomal gel on skin cancer was greater than the antitumor activity of the gel preparation containing free metformin. Lower lecithin, high ethanol and isopropyl alcohol and moderate cholesterol contents improved the permeation rate. Overall, we can conclude that metformin-loaded ethosomes are a promising remedy for treating skin cancers, and more studies are warranted to approve this activity in other animal models of skin cancers.

## 1. Introduction 

Skin cancer is a rapidly increasing malignancy affecting humans worldwide [1]. Therapeutic options for skin cancer that include vemurafenib, vismodegib and cemiplimab-rwlc have been marketed [2,3]. These drugs are promising for treating skin cancer, as they enhance the overall survival and shrinkage of the primary tumors. These therapies appear promising but cannot treat 60% of patients. Further, patients develop a high tolerance to these therapies after a few weeks of treatment, enabling metastatic growth and relapse [4]. Hence, it is very important to discover new anti-skin cancer drugs and formulations [5].

Metformin is an important antidiabetic medication [6] that reduces the glucose level, leading to a reduction in the blood insulin level; thus minimizing its impact as a tumor growth factor [7]. Adenosine monophosphate-activated protein kinase (AMPK) is an energy central regulator that plays a crucial role in the restoration of energy balance within the cell in many metabolic pathways [8]. The direct effect of metformin is an activation of AMPK leading to the mammalian target of rapamycin (mTOR) signaling inhibition [9], which, in turn, plays a role in the proliferation of cancer stem cells [7,8,10].

Metformin was reported to suppress the growth of skin cancers in vitro [10]. Some in vivo studies declared that metformin either given in drinking water [11] or administered systemically [12] can mitigate skin cancer growth in animals. A recent study co-delivered a combination of metformin and the chemotherapeutic agent doxorubicin into melanoma tumors to trigger apoptosis and necrosis of the melanoma cells, leading to mitigation of the progression of melanoma growth [13]. One recent clinical trial showed that metformin provided a chemoprotective effect for patients at a high risk of basal cell carcinoma [14]. Additionally, metformin can inhibit skin cancer progression by other mechanisms, such as immune system activation, [15] increasing in autophagy and cell apoptosis by p53 and p21 activation, [16] inhibiting protein synthesis [17] and the destruction of cancer stem cells [14].

The oral administration of metformin produces adverse effects such as nausea, diarrhea and gastric upset, and some types of hepatotoxicity and pancreatitis have been reported. In addition, metformin is known to produce vitamin B12 deficiency, and lactic acidosis is also observed in patients with renal insufficiency [18]. Hence, the topical route should be preferred whenever appropriate. 

There are many advantages in using transdermal drug delivery systems. For example, medications can avoid hepatic first-pass metabolism and factors that modify pharmacokinetics in the gastrointestinal tract; this can improve the systemic bioavailability while also lowering the risk of drug concentration-related side effects. Since the topically applied drugs are released in a predetermined range over a long time, this often increases patient compliance, because it is simple and convenient to apply them with a low-dose frequency [19]. Moreover, the topical route provides a large and varied surface of application, as well as ease of self-administration, and is an available alternative to both oral delivery and hypodermic drug injection.

Ethosomes are lipid-based nanovesicles with improved deformability, softness and elasticity and are the most investigated vesicular system. Ethosomes are multilamellar nanovesicles that are made up of phospholipid and ethanol [20,21]. Ethanol amends the phospholipid bilayer fluidity, breaks down the membrane barrier of the stratum corneum and, hence, improves the power of penetration [21]. Ethanol is a powerful penetration enhancer that gives vesicles special characteristics such as entrapment efficacy, size, negative electric potential, stability and better skin permeability [22]. The hair follicles and stratum corneum route allow ethosomes to permeate the epidermis, and the ethosomes are released into the upper skin layer, which results in the drug substances gradually penetrating while the phospholipids stay in the upper layer of the epidermis [23].

Selecting the type and concentration of phospholipid are important to prepare ethosomes, as they will affect the size of the vesicle, stability, percent of entrapment efficacy (EE%), electric potential, drug release % (DR%) and penetration of the vesicles into the skin [21]. Cholesterol is a rigid steroid molecule that enhances the drug stability and entrapment efficiency when used in ethosomal systems [24]. Isopropyl alcohol has a significant impact on the entrapment efficiency but a minor impact on DR% according to the transdermal drug–flux measurements through mice skin [25]. 

The current study aimed to formulate and characterize metformin-loaded ethosomal preparations and to select the best optimal formula to test its topical antitumor activity against experimentally induced skin cancer in mice. This study also aimed to deliver metformin to the skin layers for the treatment of skin cancer.

## 2. Results

The effects of different variables such as phospholipid, cholesterol and ethanol were evaluated through the evaluation of EE%, particle size, Zeta potential (ZP), vesicle size (VS) and DR%. Table 1 demonstrates the results of the 17 experiments.

### 2.1. Influence of the Independent Variables on Entrapment Efficiency % 

Table 1 shows the data of EE% for all the prepared formulations. The model obtained a suitable fitting with a linear model, the calculated correlation coefficient (R^2^) was 0.8388 and the predicted R^2^ was 0.6639, while the adjusted R^2^, which was 0.7948. The difference was less than 0.2. The ANOVA obtained a significant difference *(p* < 0.0001) between the preparations. Equation (1) describes the influence of the independent variables on the EE%, as follows:

EE% = +98.13 − 0.15 A + 0.0037 B + 0.1062 C(1)

where A= lecithin, B = cholesterol and C = ethanol.

**Figure 1:**
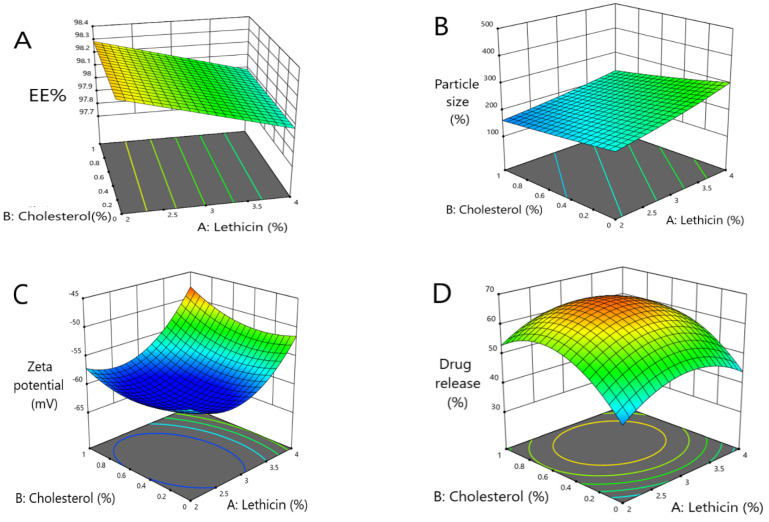
Three-dimensional response surface plots presenting the influence of the independent variables on (**A**) the EE%, (**B**) vesicles size, (**C**) ZP and (**D**) percent of drug released after 8 h.

**Figure 2:**
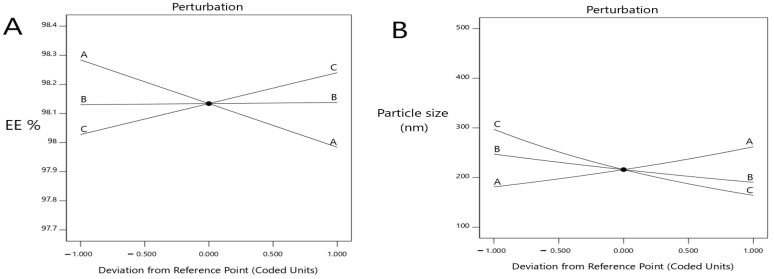

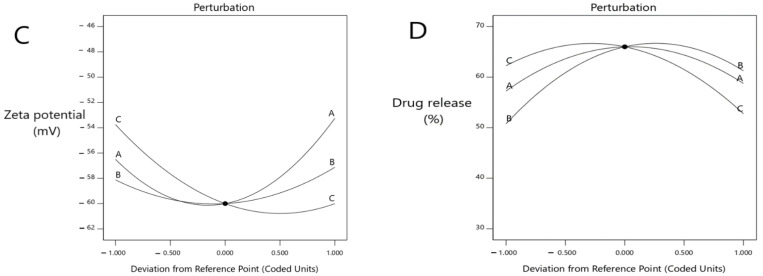
Perturbation explains the influence of the independent variables at (**A**) the EE%, (**B**) vesicles size, (**C**) ZP (mV) and (**D**) percent of drug release after 8 h.

As shown in Figure 1, when the concentration of lecithin increased, the EE% of metformin decreased with a significant difference (*p* < 0.0001), where the EE% of formulas #6 and #11 were 98.14 ± 0.92 and 98.08 ± 0.5, respectively. Further, an increment in the concentration of cholesterol led to an increased EE% of metformin (*p* < 0.05), where the EE% of formulas #6 and #9 were 98 ± 1.2% and 99.4 ± 0.24, respectively. When the ethanol concentration increased, the EE% of metformin increased (*p* < 0.033), where the EE% of formulas #9 and #13 were 99.4 ± 0.24 and 98.4 ± 0.35, respectively (Figure 1A and Figure 2A).

### 2.2. Influence of the Independent Variables on the Vesicle Sizes

Data of the vesicle sizes for all the formulations are demonstrated in Table 1. The model obtained a suitable fitting with a linear interaction among the variables, and the correlation factor (R^2^) was 0.8560. The ANOVA testing obtained a significant difference (*p* < 0.0001) between 0.0689 ± 0.0042 nm and the suggested equation (Inverse Sqrt transform), as follows:

1/Sqrt (particle size − 1.00) = +0.0528 − 0.0686 A − 0.0045 B + 0.0101 C(2)

As shown herein, by increasing the concentration of lecithin, the ethosomes vesicle size increased (*p* < 0.0025) where the vesicle sizes of formulas #17 and #5 were 380 ± 45 nm and 223 ± 9 nm, respectively. Further, by increasing the concentration of cholesterol, the ethosomes vesicle sizes increased (*p* < 0.0306); the vesicle sizes of formulas #16 and #6 were 234 ± 20.6 nm and 203 ± 11.3 nm, respectively. By increasing the concentration of ethanol, the ethosomes vesicle size decreased (*p* < 0.0001); the vesicle sizes of formulas #13 and #9 were 124 ± 14.2 nm and 161 ± 13.2 nm, respectively (Figure 1B and Figure 2B). 

### 2.3. Influence of the Independent Variables on ZP

Table 1 shows the ZP values for all the prepared formulations. The model obtained a quadratic equation, and the correlation factor (R^2^) was 0.9935. The ANOVA test highlighted a significant difference in the ZP at *p* < 0.05, and the suggested equation was follows:

ZP = −60 +1.62 A + 0.5 B − 3.13 C + 1 AB + 3.25 AC + 0.5 BC + 5.13 A^2^ + 2.38 B^2^ + 3.12 C^2^ (3)

The ZP decreased with the increasing lecithin concentration, until a specific point, then increased the lecithin percentage from 2% to around 2.82%, which led to a decrease in ZP. When the lecithin percentage increased more than 2.82%, the ZP increased again (significant difference, *p* < 0.0001), where the ZP of formulas #13 and #17 were −60 ± 1.4 mv and −60 ± 1.4 mv, respectively. The ZP decreased as the cholesterol concentration increased until a specific point. Then, an increased cholesterol percentage from 0 to around 0.5 led to a decline in the ZP, and when the cholesterol percentage increased more than 0.5, this led to an increase in the ZP (*p* < 0.0413), where the ZP of formulas #13 and #6 were −60 ± 1.4 mv and −49 ± 0.8 mv, respectively. The ZP decreased with the increasing ethanol until a specific point, then increased the ethanol percentage from 20% to around 36%, which led to a decrease in the ZP, and when the ethanol percentage increased more than 36%, the ZP increased again. These results obtained a significant difference (*p* < 0.0001), where the ZP of formulas #13 and #3 were −60 ± 1.4 mv and −47 ± 1.3 mv, respectively (Figure 1C and Figure 2C).

### 2.4. Influence of the Independent Variables at a Percent of Drug Release

The DR % data of the formulations are presented in Table 1. The model revealed a suitable fitting with a quadratic model, and the correlation factor (R^2^) was 0.9870. The ANOVA highlighted a significant difference (*p* < 0.0001) at 53.53 ± 2.05%. Equation (4) describes the influence of the independent variables at the DR%:

DR% = +66 − 0.75 A − 5.25 B + 4.75 C − 0.5 AB − 9 AC − 10 BC − 8 A^2^ − 10 B^2^ − 8.5 C^2^ (4)

**Figure 3:**
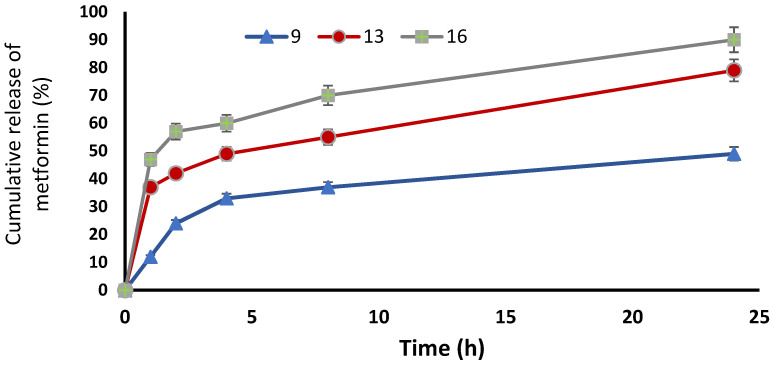
Cumulative release of metformin % from the ethosomal formulations #9, #13 and #16.

The model highlighted that the DR% significantly increased with decreasing lecithin (*p* < 0.05), while the DR% after 8 h of formulas #5 and #9 were 38 ± 0.41% and 37 ± 0.64%, respectively. In addition, the DR% increased upon decreasing the cholesterol amount significantly (*p* < 0.05), where the DR% after 8 h for formulas #5 and #9 were 38 ± 0.41% and 37 ± 0.64%, respectively. On the other hand, the DR% increased upon increasing the ethanol in a significant manner (*p* < 0.05), where the DR% after 8 h of formula numbers #13 and #11 were 55 ± 0.98% and 45 ± 0.62%, respectively (Figure 1D and Figure 2D). A free drug solution was released completely throughout the dialysis bag within 10 min. The observed rapid drug release may be explained by the sink conditions provided through the experiment. Formula #13 showed the best cumulative release of the metformin percentage from the ethosomal formulations (Figure 3).

### 2.5. Selection of the Optimized Formula

We prepared ethosomes with a high percent of entrapment efficiency, small vesicle size, high ZP and high percent of DR% by using a three-level three-factor Box–Behnken design. The ANOVA test analyzed and evaluated all the data collected from each response; then, an optimized formula was obtained using the desirability method. The formula that contained 2.083% *w*/*w* lecithin, 0.524% *w*/*w* cholesterol and 37.495% *v/v* ethanol was selected as the optimized formula, as it showed the best desirability index value (0.811). 

The chosen optimal formula, #13, displayed an EE% of 98.40 ± 0.35%, a vesicle size equal to 124.01 ± 14.27 nm and a release % equal to 55.04 ± 0.98 %. The ZP of the optimized formula #13 was 60.08 ± 1.44 mV, which provided good stability. 

### 2.6. In Vitro Studies to Evaluate Skin Permeation

**Figure 4:**
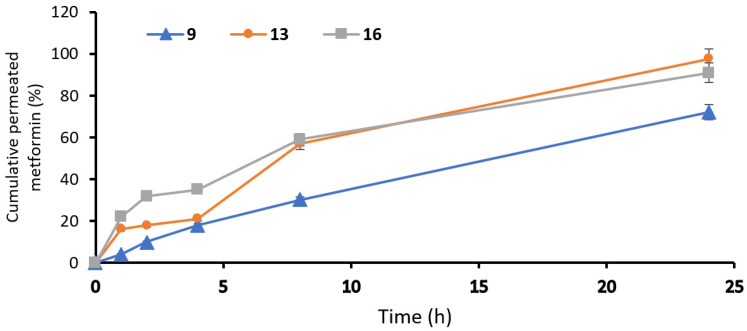
Permeation profiles of metformin from ethosomal formulations: #9, #13 and #16 ethosomal formulations.

In formula #9, the amount of permeated metformin was 1224.27 ± 18.1 µg/cm^2^, and the steady-state flux was 2.93 µg/cm^2^/h, while the percent of cumulative permeation was 72%. In the optimal formula, #13 showed an amount of permeated metformin equal to 1660 ± 32.4 µg/cm^2^, while the steady-state flux was 3.61 µg/cm^2^/h; however, the percent of cumulative permeation was 97.6%. In addition, formula #16 showed an amount of permeated metformin equal to 1547 ± 21.7 µg/cm^2^, the steady-state flux was 3.26 µg/cm^2^/h and the percent of cumulative permeation was 91%. Finally, the optimal formula #13 showed the best permeability at interval times with significance (*p* < 0.05), as this formulation had the highest ethanol and isopropyl alcohol concentration, lower lecithin concentration and moderate concentration of cholesterol (Table 2) (Figure 4). The TEER results of the measured electrostatic repulsion were above 30 ± 1.5 kΩ. That indicated a good state for the skin integrity [26].

### 2.7. Morphological Characterization of the Ethosomes

**Figure 5:**
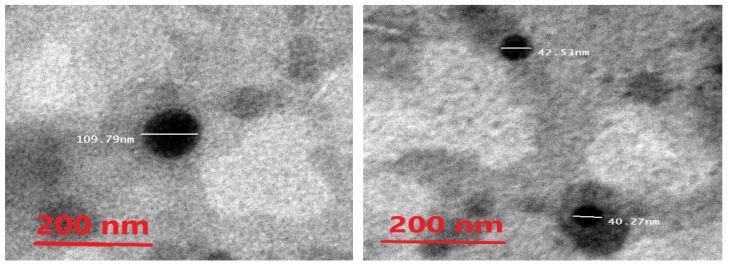
Photomicrograph of the optimal metformin-loaded ethosome formula as seen by the TEM.

The morphology of the ethosomes was characterized by using a transmission electron microscope. The optimal formula was freshly prepared, then used for the transmission electron microscopy (TEM) images. The ethosomes showed in the TEM images as black dots (Figure 5). The TEM images showed ethosomes in well-identified spherical shapes and homogenous and non-aggregated vesicles, which confirmed their nanovesicular characteristics for the ethosomes.

### 2.8. Thermal Analysis of Optimal Metformin-Loaded Ethosomes Formula 

**Figure 6:**
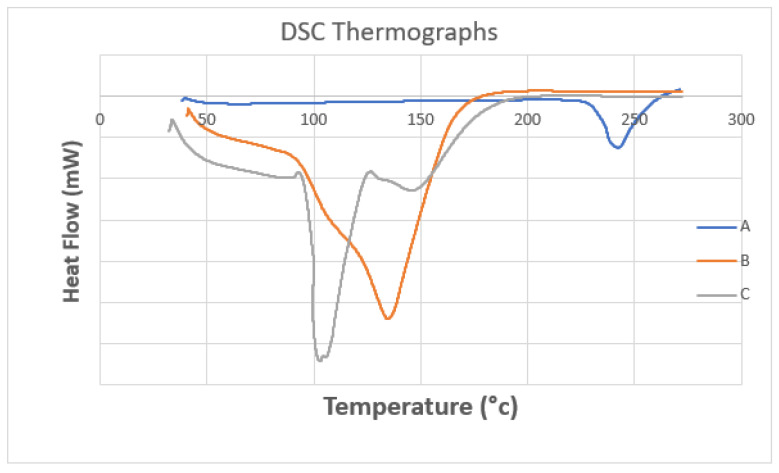
DSC thermograms of (**A**) pure metformin, (**B**) the optimal metformin-loaded ethosome formula and (**C**) an empty formula (excipient).

The pure metformin curve revealed a sharp endothermic peak at 242 °C, while the optimal metformin-loaded ethosome formula (#13) showed a peak appearing at 135 °C, but the thermogram of the empty formula (excipient) revealed two endothermic peaks at 103 °C and 148 °C. Metformin in the optimal metformin-loaded ethosome formula (formula #13) did not show a characteristic peak. These findings highlight that metformin was dissolved within the ethosomes during the formulation process (Figure 6) [27].

### 2.9. In Vivo Antitumoral Activity of the Optimized Metformin-Loaded Ethosomal Gel 

**Figure 7:**
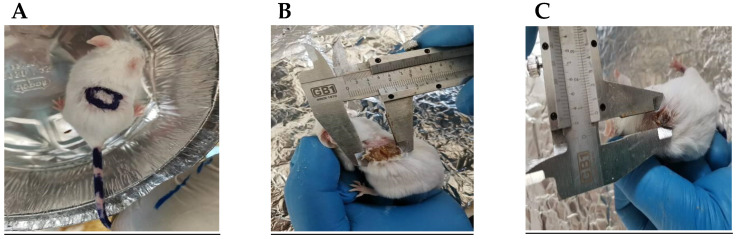
The skin lesions induced by DMBA in male mice. (**A**) A sample of the lesions in the vehicle group. (**B**,**C**) Measuring the dimensions of the lesions in a mouse from the DMBA + vehicle group. Dimensions were measured by a caliber adjusted at the edges of the lesion.

The developed 7,12-dimethylbenz[α]-anthracene (DMBA)- induced lesions appeared at the back of each mouse and were monitored weekly, as shown in Figure 7. A specialized caliber was utilized to measure the width and length of each lesion (Figure 7A,B).

#### 2.9.1. The Body Weight and Lesion Length and Width

**Figure 8:**
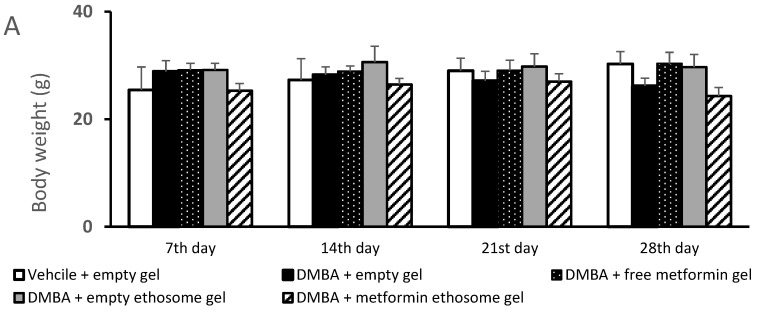

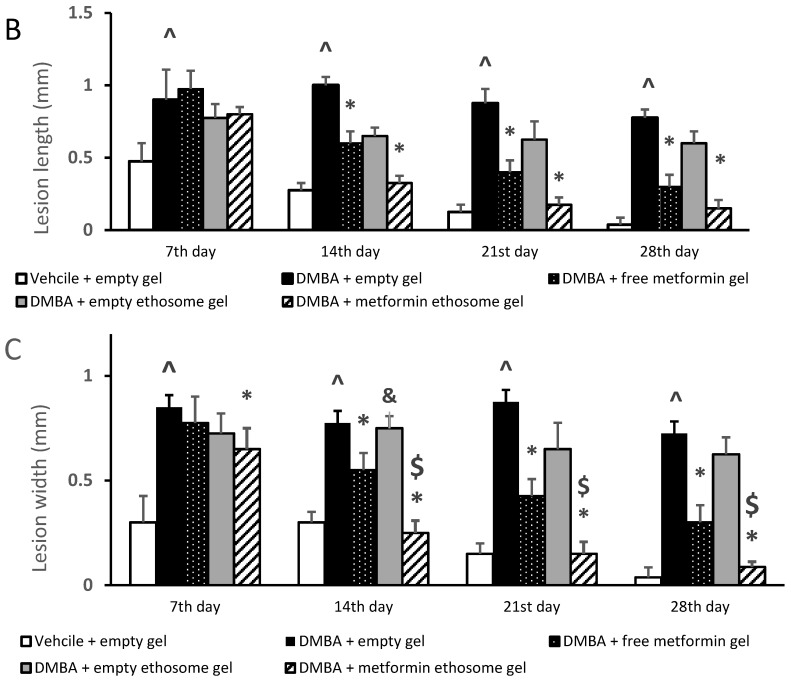
Body weights and dimensions of the skin lesions in the experimental groups. (**A**) Mice body weights, (**B**) lesion lengths (mm) and (**C**) lesion widths (mm). Data were the mean ± SD and analyzed using one-way ANOVA, followed by Bonferroni’s pairwise comparison test (*p* < 0.05). ^: versus the vehicle + empty gel group, *: versus the DMBA + empty gel group, &: versus DMBA + free metformin gel. $: versus DMBA + empty ethosome gel.

The weight of the mice and diameters of the lesions were measured to evaluate the skin cancer progression [28]. The antitumor efficacy of the metformin-loaded ethosomes was evaluated in mice group #5. This metformin-loaded ethosome-containing gel produced a significant decrease in the lesion diameters compared with the other four gels over 28 days (Figure 8).

Figure 8A demonstrates the body weights of the mice during the course of the experiment. The ANOVA indicated no significant variations among the experimental groups at the four time points. Figure 8B illustrates the gross length of the skin lesions, and we found a significant increase in the length in the DMBA + empty gel group versus the vehicle + empty gel group at the four studied time points. Mice treated with free metformin gel or metformin ethosome gel showed significantly lower lesion lengths compared to the mice treated with the empty gel (Figure 8B). In Figure 8C, the lesion width in the DMBA + empty gel group was greater than that measured in the vehicle + empty gel group. The mice group treated with DMBA + metformin ethosome gel showed a smaller lesion width at all the study time points compared to the DMBA + empty gel group. Although the mice treated with DMBA + free metformin gel showed significantly smaller lesion widths compared to the DMBA + empty gel group at day 14, day 21 and day 28, the curative effect shown in the DMBA + metformin ethosome gel group was significantly enhanced (Figure 8C). 

#### 2.9.2. The Thickness of the Hematoxylin and Eosin-Stained Skin Layers

**Figure 9:**
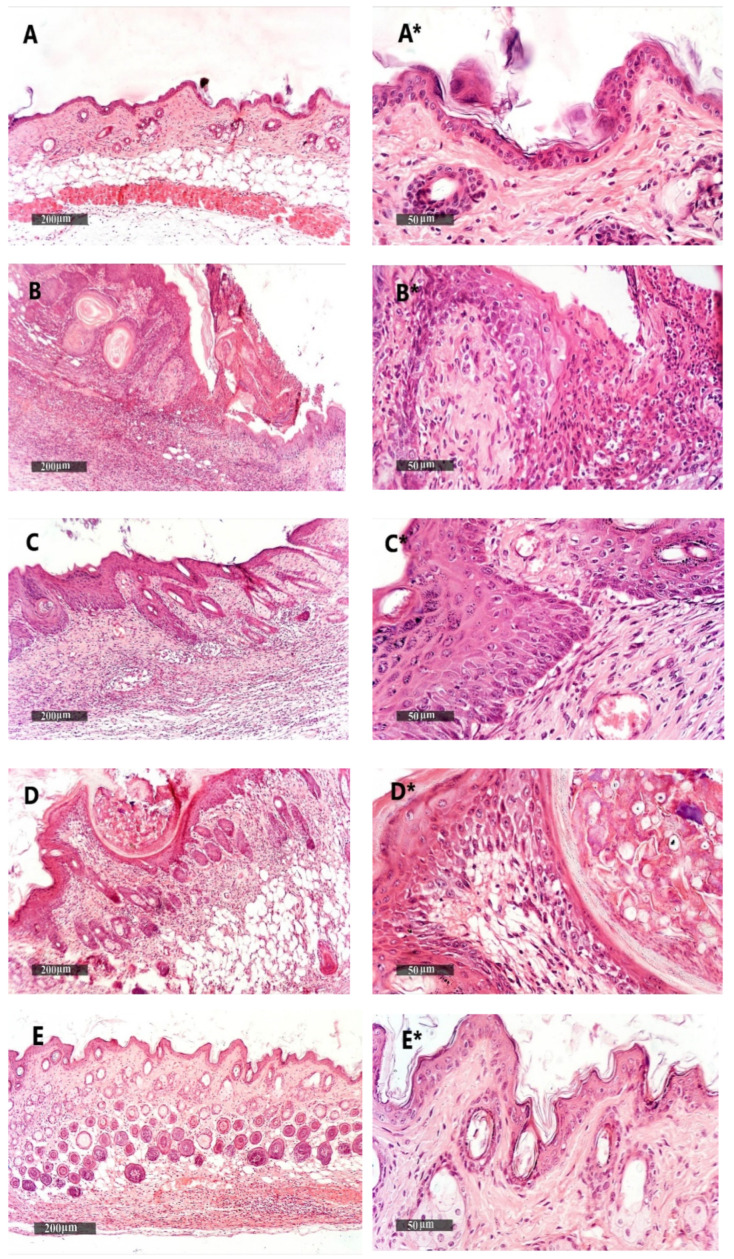

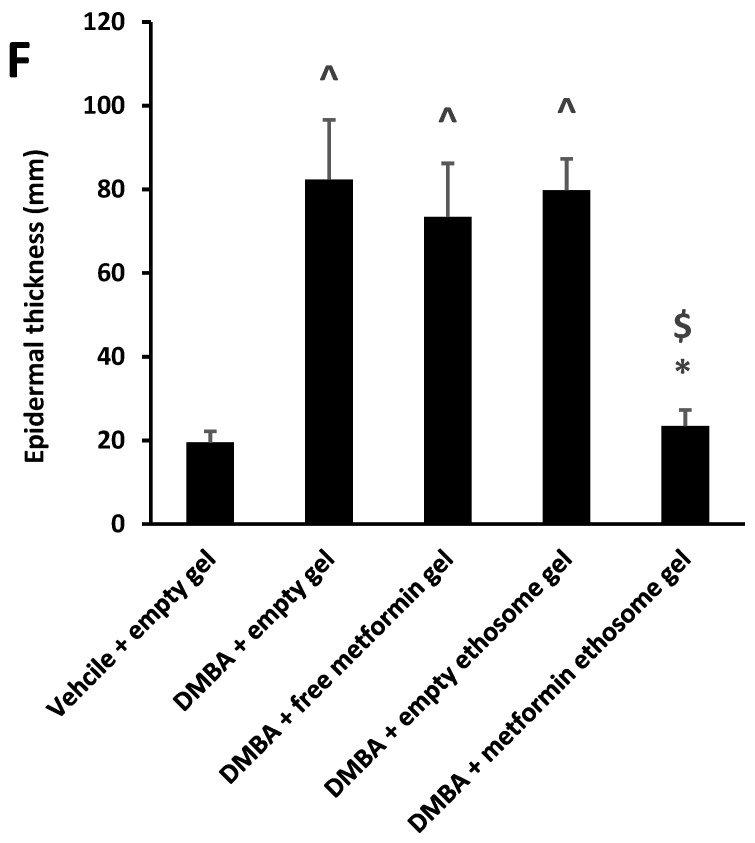
Histopathological picture of skin tissues stained with hematoxylin and eosin. (**A**,A*) Images revealed normal morphological features of mouse skin layers with an apparent intact thin epidermal layer with intact keratinocytes and an intact dermal layer with well-organized collagen fibres and hair follicles without abnormal inflammatory cells infiltrates, as well as an intact subcutaneous tissue. (**B**,B*) Images for DMBA + empty gel, showing a circumscribed raised, folded mass with non-keratinized epidermal layers of hyperplasia with the adjacent ulcerated area covered with the scab of necrotic tissue depress. The underlying dermal layer showed severe diffuse inflammatory cell infiltrates from different populations accompanied with fibroblastic activation and few records of keratin cysts, as well as abundant records of congested blood vessels (BVs). (**C**,C*) Images from the DMBA + free metformin group showing focal areas of moderate hyperplasia of non-keratinized epidermal layers without ulcerated lesion records associated with focal subepidermal fibroblastic activation with higher collagen fibres contents. The persistence of moderate-to-severe inflammatory cell infiltrates was shown; however, a less intense density compared with the model samples was accompanied by abundant records of subepidermal and subcutaneous-congested BVs. (**D**,D*) Images for skin specimens from the DMBA + empty ethosome group showing a circumscribed, raised non-ulcerated mass of skin with moderate epidermal thickening and folding covered with a mass of necrotic tissue to depress with focal subepidermal hemorrhagic patches and mild records of infiltrate of inflammatory cells, as well as fibroblastic activity with minimal records of congested subcutaneous BVs. (**E**,E*) Images for the DMBA + metformin-loaded ethosome group (optimal gel formula) showing obvious improvement of the histological organization epidermal/dermal layers with an almost apparent intact, mildly folded epidermal layer with apparent intact keratinocytes and an abundant mature collagen formation in a mildly thick dermal layer with minimal inflammatory cells infiltrates. However, there were also focal records of subcutaneous hemorrhagic patches with moderate inflammatory cell infiltrates, as well as congested and dilated BVs. (**F**) Epidermal thickness of the skin layers in all groups. Thicknesses of six regularly spaced skin parts were measured using ImageJ software (NIH, Bethesda, MD, USA). The average of the measured parts was calculated for every tissue specimen, and the mean of each group was then estimated. Data were the mean ± SD and analyzed using one-way ANOVA, while the pairwise comparison was estimated by Bonferroni’s test at *p* < 0.05. ^: versus the vehicle + empty gel group, *: versus the DMBA + empty gel group, &: versus DMBA + free metformin gel. $: versus DMBA + empty ethosome gel, scale bar = 200 μm (left column) and scale bar = 50 μm (right column).

We found that the vehicle + empty gel group displayed the normal morphological features of the mouse skin layers, with an apparent intact thin epidermal layer with intact keratinocytes and an intact dermal layer with well-organized collagen fibres and hair follicles without abnormal inflammatory cells infiltrates, as well as intact subcutaneous tissue (Figure 9A,A*). The DMBA + empty gel group showed a circumscribed raised, folded mass with non-keratinized epidermal layers hyperplasia with the adjacent ulcerated area covered with the scab of necrotic tissue depress; the underlying dermal layer showed severe diffuse inflammatory cell infiltrates from different populations accompanied with fibroblastic activation and a few records of keratin cysts, as well as abundant records of congested blood vessels (BVs) (Figure 9B,B*). Images from the DMBA + free metformin gel showed focal areas of moderate hyperplasia of non-keratinized epidermal layers without ulcerated lesion records associated with focal subepidermal fibroblastic activation with higher collagen fibres contents. The persistence of moderate-to-severe inflammatory cell infiltrates was shown, however, to have a less intense density compared with the model samples, accompanied with abundant records of subepidermal and subcutaneous congested BVs. The third group obtained healing in the damage of the epidermis and dermis layers and a decrease in the infiltrations of inflammatory cells (Figure 9C,C*). Images of the skin specimens from the DMBA + ethosome gel group showed circumscribed, raised non-ulcerated masses of skin with moderate epidermal thickening and folding covered with a mass of necrotic tissue to depress with focal subepidermal hemorrhagic patches and mild records of infiltrate of inflammatory cells, as well as fibroblastic activity with minimal records of congested subcutaneous BVs (Figure 9D,D*). Images of DMBA + metformin ethosome gel showing obvious improvements of histological organization epidermal/dermal layers with an almost apparent intact, mildly folded epidermal layer with apparent intact keratinocytes and abundant mature collagen formation in a mildly thick dermal layer with minimal inflammatory cells infiltrates. However, focal records of subcutaneous hemorrhagic patches with moderate inflammatory cell infiltrates, as well as congested and dilated BVs, were shown (Figure 9E,E*). In Figure 9, the DMBA + metformin-loaded ethosomes group showed an approximately normal structure in the epidermis and dermis layers and the nonexistence of inflammatory cells. The measured epidermal thickness was significantly decreased compared with the DMBA + empty gel group (Figure 9F).

#### 2.9.3. Histopathological Examination of Kidney Specimens Stained with H&E

**Figure 10:**
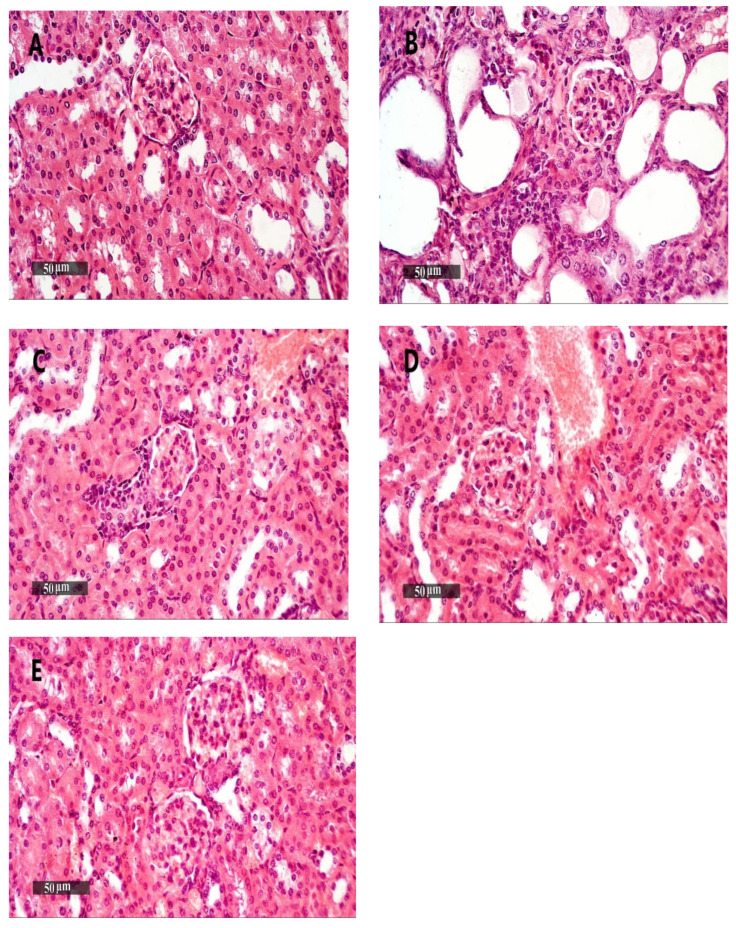
Kidney samples stained with H&E. (**A**) An image for a kidney specimen from the vehicle + empty gel group showing apparent intact morphological features of renal parenchyma, including renal corpuscles and different nephron tubular segments, including tubular epithelium, with intact interstitial tissue, as well as vasculatures. (**B**,**D**) Images for a kidney specimen from the DMBA + empty gel or DMBA + ethosome gel showing a mild cystic dilatation of renal tubular segments, with some flattening of the tubular epithelial lining. (**C**) An image for a kidney specimen from the DMBA + free metformin gel group showing mild focal records of tubular degenerative changes with intact renal corpuscles, as well as interstitial tissue with very few sporadic inflammatory cell infiltrates. (**E**) An image from DMBA + metformin ethosome gel samples showing little focal tubular degenerative changes with intact renal corpuscles, interstitial tissue and vasculatures, scale bar = 50 μm.

The kidney samples from the vehicle + empty gel group demonstrated intact morphological features of renal parenchyma, including renal corpuscles and different nephron tubular segments, including tubular epithelium, with intact interstitial tissue, as well as vasculatures (Figure 10A). DMBA + empty gel or DMBA + ethosome gel showed a mild cystic dilatation of the renal tubular segments, accompanied by little interstitial mononuclear inflammatory cell infiltrates (Figure 10B,D). The DMBA + free metformin gel group showed mild focal records of tubular degenerative changes with intact renal corpuscles, as well as interstitial tissues with few sporadic inflammatory cell infiltrates (Figure 10C). The DMBA + metformin ethosome gel samples showed sporadic records of tubular degenerative changes with intact renal corpuscles, interstitial tissue and vasculatures (Figure 10E).

#### 2.9.4. Histopathological Examination of Liver Specimens Stained with H&E

**Figure 11:**
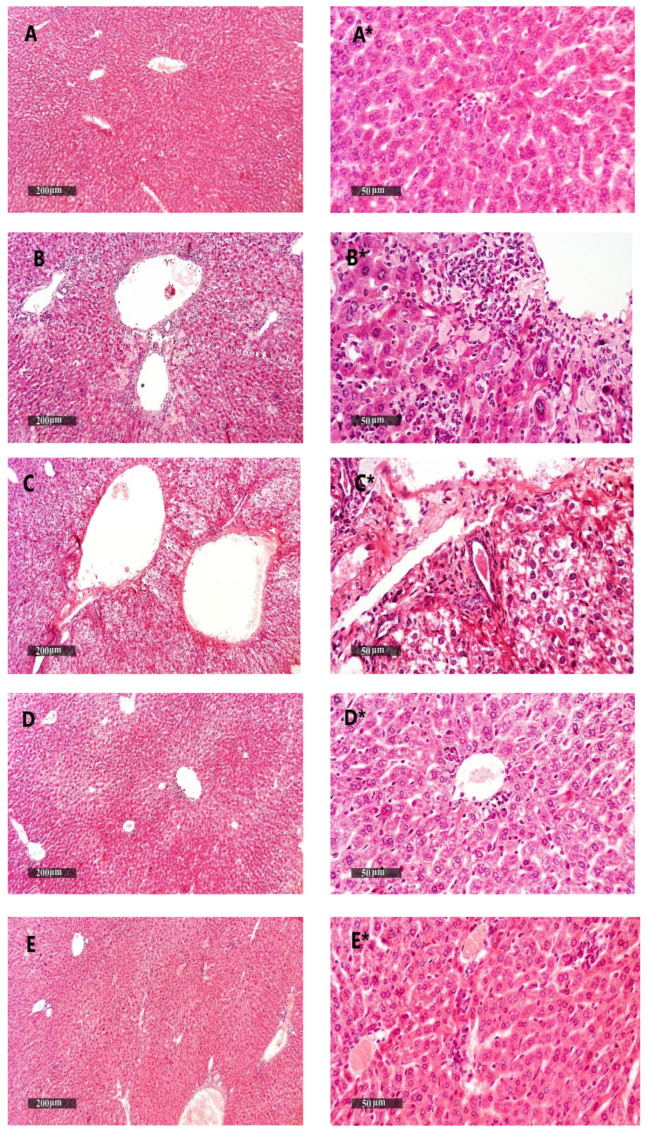
Liver samples stained with H&E. (**A**,A*) Liver samples from the vehicle + empty gel group show the normal morphological structure of hepatic parenchyma. (**B**,B*) DMBA + empty gel samples show mild hepatocellular degenerative changes in the pericentral, as well as periportal, regions, with some mononuclear inflammatory cells infiltrating in the hepatic parenchyma. (**C**,C*) Samples from the DMBA + free metformin gel group showing little hepatocellular vacuolar degenerative changes with minimal inflammatory cell infiltrate records. (**D**,D*) Samples from the DMBA + ethosome gel group showing mild records of the hepatocellular degenerative changes with higher records of apparent intact hepatocytes, and mild focal mononuclear inflammatory cell infiltrates accompanied with the mild dilatation of hepatic BVs. (**E**,E*) Samples from the DMBA + metformin ethosome gel group showing almost apparent intact hepatocytes all over hepatic parenchyma, and the moderate dilation of portal BVs with minor focal perivascular inflammatory cell infiltrates, scale bar = 200 μm (left column) and scale bar = 50 μm (right column).

Liver samples from the vehicle + empty gel group showed the normal morphological structure of hepatic parenchyma (Figure 11A,A*). The DMBA + empty gel samples showed mild hepatocellular degenerative changes in the pericentral, as well as periportal, regions with diffuse mononuclear inflammatory cells infiltrating in the hepatic parenchyma (Figure 11B,B*). Samples from the DMBA + free metformin gel group showed mild hepatocellular vacuolar degenerative changes with the dilatation of hepatic BVs and minimal inflammatory cell infiltrates (Figure 11C,C*). Samples from the DMBA + ethosome gel group showed mild hepatocellular degenerative changes with intact hepatocytes and mild focal pericentral and periportal mononuclear inflammatory cells infiltrates (Figure 11D,D*). Samples from the DMBA + metformin ethosome gel group showed almost apparent intact hepatocytes all over the hepatic parenchyma and moderate dilation of the portal BVs with minor focal perivascular inflammatory cell infiltrates (Figure 11E,E*). 

#### 2.9.5. Liver and Kidney Function Tests

**Figure 12:**
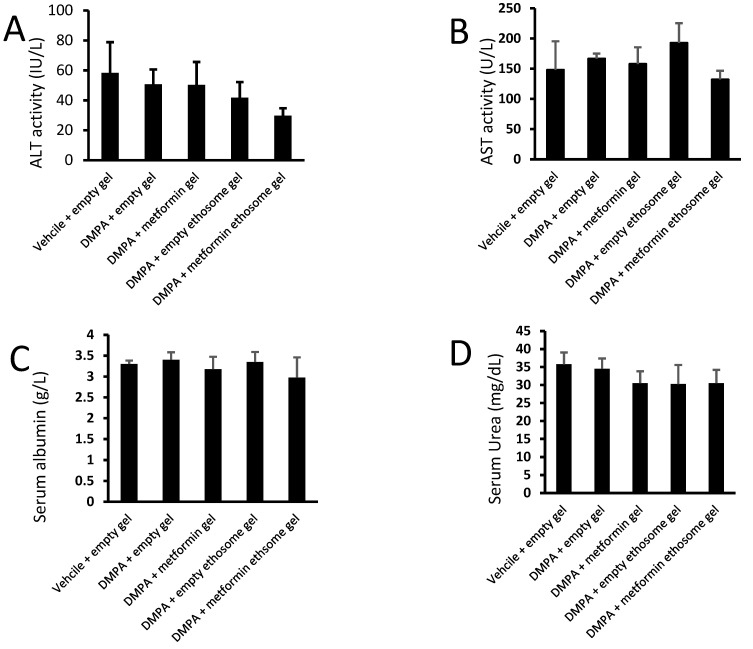

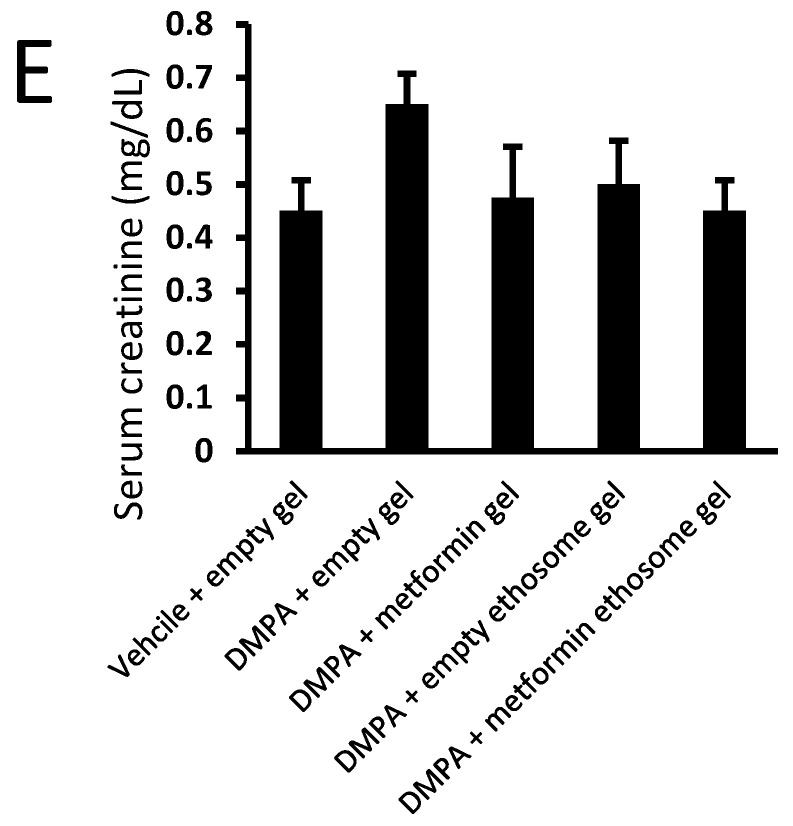
Liver and kidney function tests for mice from the experimental groups: (**A**) ALT, (**B**) AST, (**C**) albumin, (**D**) urea and (**E**) creatinine. Data were the mean ± SD, and the analysis was done by one-way ANOVA at *p* < 0.05.

We applied an ANOVA test on the data of the serum ALT, AST, albumin, urea and creatinine, but the data indicated nonsignificant differences among the study group (Figure 12A–E).

## 3. Discussion

This study aimed to obtain the ability of ethosomes to raise the retained amount of metformin on the skin to improve skin cancer treatment. Ethosomes can penetrate the stratum corneum to the deep layers, as ethosomes contain a high alcohol content [29]. 

### 3.1. Influence of the Independent Variables on EE%

The concentrations of lecithin, ethanol and cholesterol are critical parameters to prepare ethosomes [20,30]. Lecithin builds lipid bilayer membranes in multilamellar vesicles of ethosomes [31]. Cholesterol is responsible for the stability and EE% of metformin [32]. 

Ethanol gives the vesicles more freedom and stability by providing softness and a negative charge [32]. Depending on the data collected from all formulations, a ethanol concentration of 40% was suitable to prepare the ethosomes that produced a high EE% and permeation [21,28,32]. However, increasing the concentration of ethanol above 40% will dissolve the ethosome membranes, causing a decrease in the EE% and increase in the vesicle sizes [32]. On the other hand, isopropyl alcohol is used with ethanol to prepare ethosomes as a skin penetration enhancer and to increase the EE% [25]. The high entrapment efficiency of the formulations is due to adding isopropyl alcohol with ethanol; isopropyl alcohol decreases the vesicle size and increases the ZP and EE%. Isopropyl alcohol can release metformin in the long term, which achieves the goal of our study [25,28,33]. 

Lecithin builds a phospholipid structure and is responsible for a multilamellar membrane of ethosomes [32]. Cholesterol has an important role in preventing leakage and reducing the permeability of drugs from vesicles [34]. Additionally, when the concentration of lecithin increases, this will lead to reducing the EE% because of the hydrophilic nature of metformin [35]. 

### 3.2. Influence of the Independent Variables on Vesicle Size and ZP

A concentration of 2–4% lecithin was used to prepare formulations of the ethosomes. Increasing the lecithin ratio will increase the vesicle sizes [36,37]. When the concentration of lecithin increases, this will lead to increasing the ethosome vesicle sizes, as lecithin molecules tend to coalesce and aggregate [38].

The negative charge of the vesicles was linked to high concentrations of ethanol, and this led to solubilizing some of the amount of lecithin inside the vesicles, which caused small multilamellar vesicles (SMLV), as ethanol has a high fluidizing effect [39]. Cholesterol had a positive effect on the vesicle size, which meant increasing the cholesterol range from 0.5% to 1%, leading to an increase in the vesicle sizes of the ethosomes [40]. 

A previous report indicated that a rise in ethanol concentration led to a decline in the ethosome vesicle sizes [41]. Ethanol plays a crucial role in skin penetration [42]. The concentration of ethosomes in most ethosomal formulations was 20–40% [37]. On the other hand, when the ethanol concentration increased, this resulted in a decline in the ethosome sizes [37]. Similarly, an incremental increase in the isopropyl alcohol concentration resulted in a decline in the ethosome vesicle sizes [25]. Due to the high value of the negative charge for the ZP, an electrostatic repulsion was formed and prohibited any aggregation between vesicles, which led to an increased stability of ethosomes [43,44].

Adding isopropyl alcohol and a high concentration of ethanol caused a high negative charge to the ethosomal formula, which led to a high penetration profile, and a high negative charge also caused a high EE%, as metformin is a cationic drug [28]. 

### 3.3. Influence of the Independent Variables on DR%

In our study, isopropyl alcohol and ethanol improved the metformin release from ethosomes, as they can increase the liquefaction and permeability that leads to an increased DR% [45]. Cholesterol and lecithin decrease the metformin release from ethosomes, as increasing concentrations of cholesterol and lecithin are incompatible with metformin solubility. Lecithin has a negative effect on DR%, as increasing the lecithin level will cause an increase in the vesicle rigidity and will cause a decrease in the DR% [46]. 

The optimal formula #13 showed the best DR% at the interval times, as this formulation has the highest ethanol and isopropyl concentration, lower lecithin concentration and moderate concentration from cholesterol. Using formulations with good penetrating and releasing properties produced an acceptable impression for the induction of a sustained releasing state.

### 3.4. In Vitro Skin Permeation Study

In our formulations, when the concentration of lecithin decreased, the permeation rate of metformin increased. Furthermore, decreasing the concentration of cholesterol caused an increase in the permeation rate of metformin. Similarly, one previous study found that, when the concentrations of lecithin and cholesterol increased, the rigidity of the ethosomal vesicle bilayer increased [32]. Ethanol enhanced the permeation rate of the drug as it interacted with the polar head group of the SC lipid molecules, lowering the melting point of the SC lipids and thereby increasing the lipid bilayer fluidity and cell membrane permeability [22,47]. The maximum permeability of the drug from the vesicles was due to a synergistic mechanism involving ethanol, vesicles and SC lipid molecules [29,48].

Carbopol has an anionic polymer and the best buffering capacity characters that keep the required pH and prevent skin irritation. When carbopol is mixed with ethosomes, it leads to reaching the required viscosity and the best bio-adhesion characteristics [49,50].

### 3.5. Thermal Analysis of Optimal Metformin-Loaded Ethosomes Formula

DSC has been used to show the physical state of metformin within the ethosomes, as well as the intermolecular interactions between metformin and ethosomes [27]. There was a lack for the characteristic metformin peaks, showing that metformin was fully solubilized in the ethosome system. On other hand, metformin was present in an amorphous state in the ethosomes; this can improve the release of the drug from the vesicles. The excipients and metformin showed no incompatibility state because of the absence of the characteristic melting point peak of the drug in the optimal formula [51].

### 3.6. In Vivo Antitumor Activity and Toxicology

The application of metformin-loaded ethosomes showed significant antitumor activity against the skin cancer compared to the application of free metformin. At the 14-days treatment point, the effect of the free metformin gel was better than the empty ethosome gel; this may be linked to the anticancer effect of the free metformin. However, at the following time points (day 21 and day 28), there was no significant differences among the two groups. 

In agreement with our study, a recent study highlighted that the application of metformin inhibits the promotion of experimental skin tumors in mice [52]. Several previous studies indicated that ethosomal preparations enhance topical anticancer drugs; for example, one research group documented that a gel preparation containing sonidegib-loaded ethosomes produced desirable therapeutic profits for treating skin cancer [28]. Another study indicated that ethosomes coloaded with evodiamine and berberine chloride showed a greater efficacy in the treatment of melanoma compared to the free forms [53]. Consistently, one more study confirmed that topically applied positively charged ethosomes of vismodegib was promising for the effective treatment of basal cell carcinoma, with a low incidence of adverse effects [28]. Regarding the toxicology study, we found that the liver and kidney histopathological changes were mild and did not indicate significant damages that would be reflected in a significant rise in the serum hepatic enzymes or kidney markers. 

## 4. Material and Methods

### 4.1. Materials

Metformin hydrochloride (99.45% powder, BP 2012), 99.9% ethanol (*v/v*), isopropyl alcohol and carbopol 974p were obtained from Medical Union Pharmaceuticals (Ismailia, Egypt). Some 10× phosphate buffered saline was bought from Lonza Company (Verviers, Belgium). The 97% L-α-lecithin was granular, from soybean oil, CAS 8002-43-5, molecular weight = 750 g/mol and the method detection limit (MDL) number was MFCD00082428. The 97% cholesterol was bought from Acros Organics (Geel, Belgium).

### 4.2. Box–Behnken Experimental Design

By using the Box–Behnken (BB) three-level three-factor design shown in Table 3, we optimized and selected the formulation variables statistically for the preparation of ethosomes that carry metformin to achieve the maximum EE%, small vesicle size, high ZP and the greatest DR% [28]. The experimental design was generated and evaluated by the aid of the Design-Expert software (Version 11, Stat-Ease Inc., Minneapolis, MN, USA).

Seventeen experiments were prepared, and the 3 independent variables were studied: L-α-lecithin concentration (2–4 *w*/*w*%) (X1), cholesterol concentration (0 to 1 *w*/*w*%) (X2) and ethanol and isopropyl alcohol concentrations (20–40 *w*/*w*%) (X3). On other hand, the EE% (Y1: EE%), vesicle size (Y2: VS), ZP (Y3) and DR% (Y4) were chosen as the dependent variables. 

A concentration of 2–4 *w*/*w*% lecithin was used to prepare formulations of ethosomes. [36,37]. The concentration of ethosomes in most ethosomal formulations was 20–40 *w*/*w*% [37]. A concentration of 0 to 1 *w*/*w*% cholesterol was used to prepare ethosomes in the most recent researches [32,54]. The optimal formula was chosen based on its desirability, which was then subjected to further examination [39].

### 4.3. Formulation of Metformin-Loaded Ethosomes

Formulation of the metformin-carrying ethosomes was designed depending on the method reported previously [21,33,34]. The aqueous and organic phases were prepared separately. Lecithin and cholesterol were dissolved in a mixture of ethanol and isopropyl alcohol to produce the organic phase, which was kept in a closed container. Metformin was insoluble in ethanol and isopropyl alcohol, so it was dissolved in distilled water to produce the aqueous phase. The aqueous phase was added to the organic phase drop by drop by a syringe pump. The mixture is stirred using a magnetic stirrer (Intilab, Cairo, Egypt) at a speed of 700 rpm for 5–30 min to obtain the required ethosomal formula at 30 °C. Finally, the ethosomal formulations were passed through a polytetrafluoroethylene (PTFE) filter with a pore size of 0.22 μm [39]. Then, the filtrates were stored in closed containers at 4 °C.

### 4.4. Characterization of the Metformin-Loaded Ethosomes

#### 4.4.1. Determination of entrapment efficiency %

EE% is the percent of the total amount of metformin encapsulated in vesicles in the formulations. The unentrapped metformin was separated using a cooling centrifuge rotating at 16,000 rpm at 4 °C (Sigma cooling centrifuge, Sigma Laborzentrifugen GmbH, Germany) [39]. The supernatants were diluted in distilled water (10 mL, 3 min) [28]. The amount of entrapped metformin was estimated spectrophotometrically (Jasco UV–Vis spectrophotometer, Jasco, Japan), and the ƛmax of metformin was 234 nm; it was calculated using a standard calibration curve. By subtracting the amount of metformin in the supernatant from the initial amount and then dividing the result by the initial amount, the EE% can be calculated [55]:

$$EE\%=\frac{Total\;\left(50\;\mathrm{mg}\right)-Free}{Total\;\left(50\;\mathrm{mg}\right)}\times 100$$

#### 4.4.2. Vesicle Size Analysis

Vesicle size is evaluated by using the dynamic light scattering method that is performed in the Malvern Zetasizer (Nano ZS, Malvern, UK) (DLS). Distilled water was utilized to dilute all formulations and mixed by shaking before the measurements to improve the scattering intensity and remove the multiple scattering phenomena. The particle size was measured after placing the samples in glass cuvettes [56]. Three replicates were done for each formulation and presented as the mean ± SD.

#### 4.4.3. Zeta Potential Analysis

We measured the ZP using a computerized Malvern Zetasizer (Instrument at Manipal University, Manipal, India) based on the electrophoretic mobility. The particle charge is an important parameter to ensure the ethosomal suspension stability [42].

#### 4.4.4. In Vitro Release Study 

Some 1-mL samples from each formula (1.7 *w*/*w*% of metformin) were added to a dialysis bag (Mw cut-off = 14,000 Da). Forty millilitres of Sorensen phosphate buffer (pH = 6.5) was used as a release medium [39]. Then, the dialysis bag was immersed in the prepared release medium at 32 ± 0.5 °C in a dissolution apparatus (SR8, Hanson Research, Chatsworth, CA, USA) at 100 rpm. New 1-mL samples were withdrawn from the medium and replaced with the same volume from the fresh medium at 1, 2, 4, 8, 12 and 24 h. Estimation of the sample concentrations was done spectrophotometrically at a 234 nm [31,55].

$$\mathrm{DR}\%=\frac{\mathrm{The}\;\mathrm{amount}\;\mathrm{of}\;\mathrm{metformin}\;\mathrm{released}\;\mathrm{at}\;\mathrm{time}\;t}{\mathrm{The}\;\mathrm{initial}\;\mathrm{amount}\;\mathrm{of}\;\mathrm{entrapped}\;\mathrm{metformin}\;}\times 100$$

#### 4.4.5. Optimization and Experimental Model Validation

Design-Expert^®^ software chooses the model that gives statistically accepted polynomial equations [57,58]. These equations are utilized for demonstrating conclusions about each response after taking both the degree and sign of the calculated coefficients. “A positive sign indicates synergism, whereas a negative sign indicates antagonism” [31]. 

Design-Expert^®^ software presents 3D response surface plots demonstrating the relation between each factor and the resultant response. The optimization process uses the desirability index (Di) to determine the suitable level of each response with an independent variable. When Di equal 0, this means an undesirable formula, while Di equal 1 means a desirable formula [31]. 

This study aimed to select the optimal formula that achieves the maximum EE% (Y1), ZP (Y3) and DR% (Y4) and minimum vesicle size (Y2). The model is accepted in case the range of the observations from the optimal formula occurs within the prediction interval of the confirmation node. The three optimized formulations were prepared 3 times for checking the optimal formula validity. Formulas with different release patterns (highest–lowest and middle values) were tested for the permeation study (Formulas 9, 13 and 16).

### 4.5. In Vitro Skin Permeation 

The diffusible membranes were collected from abdominal rat skin in the Faculty of Pharmacy, Suez Canal University, Ismailia, Egypt. The skin of rats was used fresh, as reported previously [51]. Each diffusion membrane was mounted in a vertical diffusion cell (5 cm^2^) as a donor compartment. Sorensen phosphate buffer (40 mL, pH = 6.5) was used as a receptor compartment [28]. The diffusion membrane containing 1 mL of each formula (1.7 *w*/*w*% of metformin) was immersed in the receptor compartment, which was stirred in a water bath at 600 rpm, and the temperature equaled 37 ± 0.5 °C. After that, 1-mL samples were taken from the medium and substituted by equal volumes from the fresh medium at 1, 2, 4, 8, 12 and 24 h. Finally, the samples were measured by a spectrophotometer at a wavelength of 234 nm [39,59]. The limit of quantitation (LOQ) was 0.84 μg, while the detection range was 1–20 μg/mL.

The animal skin model could provide higher permeation results than its human counterpart [60]. For checking the skin integrity, the trans-epidermal electrical resistance (TEER) test was applied. An aqueous NaCl solution (0.9%) was used for filling the diffusion cell’s receptor, as well as the donor compartments. Each compartment was immersed in electrodes, and the resistance was determined utilizing an LCR bridge (LCR400, Thurbly Thandar Instruments, Cambridgeshire, England) at a frequency equal to 1 kHz. Various factors, including the instrument, applied frequency, resultant current, solution ionic strength and the surface area of the skin sample, are able to control the estimated resistance [26]. The standard limit value was set at 1 kΩ in the experiment.

### 4.6. Analysis of Permeation Study Data

jss is the steady-state flux that is calculated by the slope of the straight line of the cumulative amount of the permeated drug per unit area at a time (μg/cm^2^/h) [39]. Kp is the permeability coefficient of metformin from each preparation (1/cm.h) that is calculated by dividing jss by the primary metformin concentration in the donor compartment (Co).

K_p_ = j_ss_/C_o_

### 4.7. Gel Formulation

The optimum formula gel was prepared by adding 0.7 g from carbopol 974p to the optimal formula under vigorous stirring; then, trimethylamine solution (5%) was used to neutralize the mixture, which was added drop by drop until the gel was formed [28,32]. 

### 4.8. Morphological Examination of the Optimal Metformin-Loaded Ethosomes Formula

The morphology of the optimal metformin-loaded ethosome formula (#13) in the gel preparation ensured that the vesicles were still formed. The gel was characterized by using TEM (TEM-1010, Tokyo, Japan) [61]. After sample preparation, it was dropped onto the surface of a copper grid coated with carbon. Each sample was left to dry in order to permit ethosomes to adhere to the carbon substrates. For staining, we applied a drop of 1% aqueous phosphotungestic acid dye to the grid, which was then air-dried for 2 min after removing excess dye with a piece of filter paper. The TEM was then used to examine and visualize the stained sample.

### 4.9. Thermal Analysis of Optimal Metformin-Loaded Ethosomes Formula

The thermal analysis of the optimal metformin-loaded ethosomes formula was studied by utilizing differential Scanning Calorimetry (Shimadzu, DSC 60, Kyoto, Japan). Five milligrams of each sample were added to an aluminum pan. Each sample was heated from room temperature to 300 °C at a heating rate equal to 10 °C/min under nitrogen flowing at a rate of 20 mL/min to prevent oxidation of the sample [62]. Pure metformin, optimal metformin-loaded ethosome formula and empty formula (excipient) thermograms have been compared.

### 4.10. In Vivo Mouse Study for Screening of Antitumor Activity and Toxicity

#### 4.10.1. Mice Preparation and Ethical Approval

Thirty male Swiss albino mice (weight range equaled 25–30 g, 6–8 weeks of age) were purchased from VACSERA (Helwan, Egypt) and placed in groups of six in plastic cages. The experiment was done in the institutional animal house at the Faculty of Pharmacy, Suez Canal University, at a temperature range equal to 23 ± 5 °C, and the animals had free access to their normal diet and drinking water. The protocol of this study obtained approval from the institutional research ethics committee (#202004MA1).

#### 4.10.2. Induction of Skin Lesions 

A 2 × 2 cm^2^ dorsal skin area was shaved on all animals using a hair clipper 48 h prior to the experiment. To induce skin lesions in mice, one dose of DMBA, which acts as an initiator for skin tumors (100 μg in 200 μL acetone) [63], was injected subcutaneously into each mice [28]. After one week, there was an increase in the number of epidermal lesions (the lesions > 1 mm in diameter for each mouse [63]. The skin lesions were assessed first by skin morphology (lesion width and lesion length) and, also, by histological methods (thickness of the epidermis). 

#### 4.10.3. Regimen of Applying Metformin-Loaded Ethosomes

Each group contained 6 animals, and the selected optimal formula was applied topically on the dorsal region of the skin (10 mg/cm^2^ of the affected area) per week for a total of 4 weeks [27,48]. The experimental groups are shown in Table 4.

The topical empty gel contained distilled water and carbopol 974 p only. On the other hand, the topical empty ethosome gel contained all the ethosome components (distilled water, ethanol, isopropyl alcohol, lecithin and cholesterol) and carbopol 974 p without metformin.

The measurements of the diameters of the skin cancer lesions were standardized for evaluating the efficacy of the selected optimal gel formula. Each mouse lesion more than 1 mm in diameter was measured weekly until the end of the study. At the end of the study protocol, the final lesion diameters were determined, and then, the mice were anaesthetized and slaughtered [64]. Blood samples were taken by cardiac puncture and settled for 30 min before centrifugation and separation of the serum samples.

#### 4.10.4. Histopathological Methodology and Examination 

Extracted skin specimens were fixed in 10% neutral-buffered formalin, then embedded in paraffin wax and sectioned by a microtome (at 4 μm) and processed for hematoxylin and eosin (H&E) staining (the sections were deparaffinized, rehydrated in alcohol, stained in Harris hematoxylin, rinsed in 95% ethanol, counterstained with eosin solution, dehydrated through 95% alcohol and cleared in xylene, followed by mounting). Light microscopy was used to examine the skin sections by an experienced pathologist (the nuclei, nucleolus and nuclear membrane were stained blue, and the cytoplasm and connective tissue were stained pink) [30]. Histopathological investigation for skin tumor specimens was performed to assess the efficacy of the different drug/vehicle formulations [63]. Thicknesses of six regularly spaced skin parts were measured using ImageJ software (NIH, Bethesda, MD, USA). The average of the measured parts was calculated for every tissue specimen.

#### 4.10.5. Toxicological Screening 

For testing any possibility of hepatic or renal toxicity due to the systemic absorption of the gel formula, a histopathological investigation was done for the liver and kidney specimens. The tissue samples were fixed in neutral-buffered formalin and processed for H&E staining and examination under light microscopy by an experienced blinded pathologist. In addition to the histopathological examination, the serum samples were directed for estimation of the liver enzymes (ALT and AST) and serum creatinine, urea and albumin.

### 4.11. Statistical Analysis

GraphPad prism was used to apply the statistical tests to the current data. Data were quantitative in nature and demonstrated in the form of the mean ± SD and analyzed using one-way ANOVA, as one factor (treatment regimen) was influencing the study groups. Bonferroni’s test for multiple comparison analysis was at *p* < 0.05. 

## 5. Conclusions

The topical application of ethosomal gel of metformin has a significant effect on treating chemically induced skin cancer in mice. This was shown by using the Box–Behnken design of “a three-level three-factor” to present a high percent of the EE%, minimum vesicle size, maximum ZP and high DR%. 

Adding isopropyl alcohol with ethanol to form ethosomes increased the ability of ethanol to solubilize lecithin, which led to an increased stability and effectiveness of the ethosomes vesicles. Isopropyl alcohol also decreased the particle size of the vesicles, which increased the EE% and allowed the metformin to be released for an extended period. This is the goal of our research. Finally, a lower lecithin, high ethanol and isopropyl alcohol and moderate cholesterol obtained an enhancement of the permeation rate. Hence, the current findings may open up an avenue for future formulations for metformin as a therapeutic tool in fighting skin cancer. 

## Figures and Tables

**Table 1:** Effect of the independent variables on the EE%, VS, ZP and DR%.

Formula Number	Lecithin *w*/*w*% (X_1_)	Cholesterol *w*/*w*% (X_2_)	Ethanol and Isopropyl Alcohol *w*/*w*% (X_3_)	EE% (Y_1_)	VS (nm) (Y_2_)	ZP (mV) (Y_3_)	DR% (Y_4_)
1	2	0	30	98.26 ± 0.52	203.00 ± 15.07	−54.05 ± 2.35	42.07 ± 0.34
2 *	3	0.5	30	98.08 ± 0.82	200.04 ± 11.21	−60.02 ± 2.21	66.31 ± 0.52
3	2	0.5	20	98.26 ± 0.41	245.11 ± 20.52	−47.31 ± 1.33	43.45 ± 0.45
4 *	3	0.5	30	98.08 ± 0.82	200.04 ± 11.21	−60.02 ± 2.21	66.31 ± 0.52
5	4	0.5	40	98.14 ± 0.92	223.02 ± 9.01	−50.23 ± 1.44	38.06 ± 0.41
6	4	1	30	98.01 ± 1.20	203.34 ± 11.30	−49.24 ± 0.87	53.14 ± 0.23
7	3	0	20	98.44 ± 0.35	414.01 ± 55.04	−51.01 ± 0.93	38.03 ± 0.82
8	3	0	40	98.26 ± 0.40	560.01 ± 127.14	−58.03 ± 1.20	45.47 ± 0.24
9	3	1	40	99.40 ± 0.24	161.03 ± 13.23	−57.04 ± 2.37	37.28 ± 0.64
10 *	3	0.5	30	98.08 ± 0.82	200.04 ± 11.21	−60.02 ± 2.21	66.31 ± 0.52
11	4	0	30	98.08 ± 0.52	173.13 ± 18.61	−53.17 ± 2.01	45.04 ± 0.62
12 *	3	0.5	30	98.08 ± 0.82	200.04 ± 11.21	−60.02 ± 2.21	66.31 ± 0.52
13	2	0.5	40	98.40 ± 0.35	124.01 ± 14.27	−60.08 ± 1.44	55.04 ± 0.98
14 *	3	0.5	30	98.08 ± 0.82	200.04 ± 11.21	−60.02 ± 2.21	66.31 ± 0.52
15	2	1	30	98.30 ± 0.44	192.41 ± 17.30	−54.31 ± 4.28	52.41 ± 0.45
16	3	1	20	98.11 ± 0.73	234.13 ± 20.63	−52.24 ± 1.81	70.02 ± 0.45
17	4	0.5	20	97.80 ± 0.23	380.06 ± 45.09	−50.06 ± 1.22	62.16 ± 0.45

EE%: entrapment efficacy %, VS: vesicle size, ZP: zeta potential and DR%: drug release % after 8 h. * Centred points. Data presented as the mean ± SD.

**Table 2:** Skin permeation parameters after 24 h.

	The Amount of Permeated Metformin (µg/cm^2^)	The Steady-State Flux (µg/cm^2^/h)	The Percent of Cumulative Permeation (%)
Formula #9	1224.27 ± 18.1	2.93	72
The optimum formula #13	1660 ± 32.4	3.61	97.6
Formula #16	1547 ± 21.7	3.26	91

**Table 3:** Evaluation of the independent variables in the Box–Behnken design.

Factor	Levels of Independent Variables		
Independent Variables	Minimum (−1)	Moderate (0)	Maximum (+1)
X1 = L-α-Lecithin concentration (*w*/*w*%)	2%	3%	4%
X2 = Cholesterol concentration (*w*/*w*%)	0%	0.5%	1%
X3 = Ethanol and isopropyl alcohol concentration (*w*/*w*%)	20%	30%	40%
Y1 = Entrapment efficiency (%)	Maximum		
Y2 = Vesicle size (nm)	Minimum		
Y3 = Zeta Potential (mV)	Maximum		
Y4 = DR % (% of drug released after 8 h)	Maximum		

**Table 4:** Experimental groups for the in vivo mouse study.

Group 1	Received vehicle (acetone) and topical empty gel.
Group 2	Received DMBA and topical empty gel.
Group 3	Received DMBA and topical free metformin gel.
Group 4	Received DMBA and topical empty ethosomes gel.
Group 5	Received DMBA and metformin ethosomes gel.

## Data Availability

Data is contained within the article.
